# Determinants in HIV-1 Nef for enhancement of virus replication and depletion of CD4^+ ^T lymphocytes in human lymphoid tissue *ex vivo*

**DOI:** 10.1186/1742-4690-6-6

**Published:** 2009-01-15

**Authors:** Stefanie Homann, Nadine Tibroni, Ingo Baumann, Serkan Sertel, Oliver T Keppler, Oliver T Fackler

**Affiliations:** 1 Department of Virology, University of Heidelberg, Heidelberg, Germany; 2 Department of Otolaryngology, Head and Neck Surgery, University of Heidelberg, Heidelberg, Germany

## Abstract

**Background:**

HIV-1 Nef critically contributes to AIDS in part by augmenting virus titers in infected individuals. Analyzing which of Nef's activities contribute to HIV pathogenesis has been hampered by the lack of a cell culture model in which Nef exerts pronounced effects on HIV replication. The human lymphoid aggregate culture (HLAC) from tonsil maintains the cell populations and cytokine milieu found *in vivo*, supports a productive infection without exogenous stimulation, and Nef contributes to efficient HIV-1 replication as well as CD4^+ ^T cell depletion in this experimental *ex vivo*-model.

**Results:**

To identify determinants in Nef that mediate these activities, we infected HLAC with a panel of isogenic HIV-1_NL4-3 _strains that encode for well-characterized mutants of HIV-1_SF2 _Nef. Determination of HIV-1 replication revealed that enhancement of the virus spread by Nef is governed by a complex set of protein interaction surfaces. In contrast, increased CD4^+ ^T lymphocyte depletion depended on only two protein interaction surfaces in Nef that mediate either downregulation of cell surface CD4 or interaction with the NAKC signalosome. Consistently, in HLAC from 9 out of 14 donors, Nef enhanced CD4^+ ^T cell depletion in the absence of a significant effect on virus replication. Moreover, our results suggest that this Nef-dependent enhancement in depletion occurred predominately in uninfected bystander CD4^+ ^T cells.

**Conclusion:**

Our findings suggest that Nef facilitates depletion of CD4^+ ^T lymphocytes in HIV-1-infected lymphoid tissue *ex vivo *by increasing the pool of productively infected cells and by sensitizing bystander cells for killing. This ability might contribute to Nef's pathogenic potential *in vivo*.

## Background

The clinical manifestation of AIDS results from continuous replication of HIV in infected individuals that causes slow but steady decline of CD4^+ ^T lymphocytes to levels that no longer control opportunistic infections [1]. Despite our expanding knowledge on the molecular details of the multi-faceted interactions of HIV with its host, the basic question of which viral factors and cell death mechanisms contribute to the loss of CD4^+ ^T lymphocytes in HIV infected patients has not been entirely solved. Clearly, the decline of an HIV patient's CD4^+ ^T cell count is caused by the death of infected cells, but it also reflects the increased sensitivity of uninfected bystander CD4^+ ^T cells to undergo apoptosis as well as a reduced regenerative capacity [2-4]. This complex interplay is studied best *in vivo*; such analysis has, however, been limited by the lack of an infectible small animal model for AIDS. *Ex vivo*-cultures of lymphoid organs (human lymphoid histoculture, HLH) were therefore established as surrogate experimental systems. Among these, *ex vivo*-cultures of human tonsils proved particularly valuable as HIV readily replicates to high titers in these cultures that maintain cell composition and cytokine milieu of a primary target organ of *in vivo *HIV infection [5]. Studies in the tonsil HLH model have shed light on key pathogenic properties of HIV, including cell tropism and cytopathic effects in relation to coreceptor usage, productive infection of resting CD4^+ ^T cells, early host responses to HIV infection as well as viral coinfections [6-16]. Of note, Jekle *et al*. observed a rapid depletion of mostly uninfected bystander CD4^+ ^T lymphocytes in HIV-infected HLH [17]. Importantly, these effects are observed in HLH cultures even in the absence of exogenous activation, which typically complicates the interpretation of results obtained with e.g. PBMC cultures.

HLH cultures were also instrumental for the functional analysis of the HIV accessory gene product Nef. Nef, a 25–35 kDa protein, is encoded by all HIV-1/-2 and SIV strains and potently augments virus replication *in vivo *[18]. Consequently, defects in the *nef *gene lead to reduced virus replication in the host and thus delayed or aborted disease progression [19-21]. Together with the observation that Nef alone is sufficient to imprint AIDS-like phenotypes in *nef*-transgenic mice [22], these findings establish Nef as a central factor for the pathogenic potential of HIV. Molecular analyses have identified a series of host cell transport and signal transduction processes that are disturbed by Nef via its many protein interactions with cellular factors [23-26]. How exactly Nef boosts HIV spread *in vivo *has, however, remained largely unclear. This lack of knowledge is in particular due to the fact that so far no experimental *ex vivo*-cell system faithfully reflects the strong impact Nef has on virus replication *in vivo*. While dispensable for HIV replication in T cell lines, a moderate increase of virus replication is provided by Nef in cultures of isolated PBLs [27-30] and Nef also augments virus replication in cocultures of antigen presenting cells and T lymphocytes [31-34]. Since these systems only allow the monitoring of virus spread, HLH cultures were employed to additionally analyze the effects of Nef on depletion of CD4^+ ^T lymphocytes and revealed a significant contribution of Nef to both virus replication and CD4^+ ^T cell loss [8], an activity that is conserved across Nef variants from HIV-1, HIV-2 and SIV [35]. Analysis of Nef proteins from various HIV-1 strains indicated that this activity of Nef may be linked to its ability to downregulate the HIV entry receptor CD4 from the surface of infected cells [36]. Molecular determinants that govern Nef's activity in HLH cultures have not yet been identified. In this study we therefore made use of a panel of isogenic viruses that express well characterized Nef mutants [27] and determined their replication kinetics as well as their ability to deplete CD4^+ ^T lymphocytes in *ex vivo*-lymphoid tissue culture.

## Results

### Nef augments HIV-1 replication and depletion of CD4^+ ^T lymphocytes in ex vivo-tonsil cultures

We first sought to establish the overall effect Nef has on virus replication and depletion of CD4^+ ^T lymphocytes in *ex vivo*-cultures of human tonsil tissue from HIV-negative donors. Such cultures can be established as tissue blocks or in suspension as aggregates (human lymphoid aggregate cultures, HLAC) [12]. Initial parallel testing of both experimental systems gave identical results for the comparison of wt and *nef*-negative HIV-1 (HIV-1Δ*nef*) (data not shown). We also compared normalization of virus input based on amounts of viral antigen (p24) or virus infectivity (TCID_50_) and again did not observe significant differences (data not shown). We therefore employed HLAC and virus inoculum normalization by p24 ELISA for the remainder of this study. HLAC were infected one day after cell preparation with wt and Δ*nef *HIV-1 corresponding to 3 ng p24 per 2 × 10^6 ^cells. 24 h later the virus input was washed out and the HLAC maintained for 11 more days. On day 4, 8, and 12 p.i., cell culture supernatant was analyzed by p24 ELISA to quantify HIV-1 production and CD4^+ ^T cell depletion was determined by flow cytometry. Quadruplicate parallel infections were harvested for each time point.

**Figure 1:**
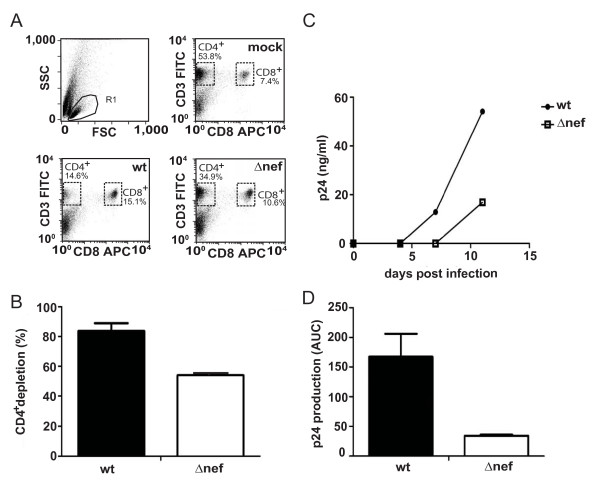
**CD4^+ ^T cell depletion and viral replication in HIV-1 wt- and Δ*nef*-infected cultures of human tonsil tissue *ex vivo***. HLACs were infected in quadruplicates with equal amounts (3 ng p24) of HIV-1 wt and Δ*nef*. Results for one such quadruplicate are shown in A and C, B and D depict mean values and SD of quadruplicate infections analyzed in parallel. (A) HLACs were stained on day 12 p.i. for CD3 and CD8 and analyzed in flow cytometry to assess numbers of CD4^+ ^and CD8^+ ^T cells. (B) CD4/CD8 ratios of infected cultures were calculated, and the percentage of CD4 T lymphocyte depletion was plotted relative to mock-infected cultures that were arbitrarily set to 0%. (C) Concentration of p24 in the culture medium over time as determined by p24 ELISA at the indicated time points. (D) p24 production over the culture period (area under the curve, AUC) of the graphs shown in C. Shown is the mean and standard deviation of all quadruplicates.

Fig. 1A presents the results of such an analysis 12 days p.i.: viable lymphocytes were identified in the FSC/SSC (gate R1) and analyzed for expression of CD3 and CD8. Direct staining of CD4 was avoided due to the reduction of CD4 surface exposure in HIV-1 infected cells, but a control staining for mock infected cells reveals that virtually all cells in this gate were positive for CD4 (see Additional file 1). A pronounced population of CD3^+^/CD8^- ^cells that represent CD4^+ ^T lymphocytes was found in mock-infected cultures (53.8% of all lymphocytes). CD8^+ ^T lymphocytes were less abundant (7.4%) and approximately 40% of all cells in the lymphocyte gate did not carry these T cell markers. In the HIV-1 wt infected culture, the CD4^+ ^population was markedly reduced to 14.6%, reflecting the strong and specific depletion of CD4^+ ^T lymphocytes due to HIV-1 replication. This CD4^+ ^T lymphocyte depletion was significantly less pronounced in the culture infected with the HIV-1Δ*nef *virus that maintained 34.9% of viable CD4^+ ^T lymphocytes. To normalize for variations in cell populations between different donors/experiments, we employed a well-established strategy [11,17,36], which determines the relative abundance of CD4^+ ^T cells by calculating the ratio of CD4^+ ^and CD8^+ ^T lymphocytes with values for mock-infected cultures set to 100%. Accordingly, average CD4^+ ^T lymphocyte depletion of 83.8 ± 5.1% and 54.2 ± 1.0% was observed in parallel quadruplicate infections of the experiment shown in Fig. 1A for HIV-1 wt- or Δ*nef*- infected HLAC, respectively (Fig. 1B). Control analyses confirmed that virtually identical degrees of CD4^+ ^T lymphocyte depletion were obtained by using the total amount of CD4^+ ^lymphocytes rather than the CD4^+ ^to CD8^+ ^ratio for evaluation (data not shown). Quantification of viral p24 antigen over the time course of infection showed that wt HIV-1 replicated more rapidly and to higher levels than HIV-1*Δnef *(Fig. 1C). In HLAC from a few donors, HIV-1 replication was overall accelerated, resulting in late peak virus production for HIV-1Δ*nef *that was comparable to that observed early in HIV-1wt infected cultures, indicating that the lack of Nef delays but not generally abrogates HIV-1 replication in HLAC (data not shown). To facilitate assessment of overall virus production during the time course of infection, the integral area under the curve (AUC) was calculated from the p24 replication kinetics plot (e.g. Fig. 1C). This evaluation accounts best for changes in p24 concentration in the cell culture medium over time [13]. Plotting of the mean AUC of the independent quadruplicates analyzed in parallel revealed that over 3-times more p24 were produced in the wt HIV-1-infected culture when compared to HIV-1*Δnef *(Fig. 1D). These results recapitulate the previously described phenotype of Nef on HIV-1 replication and CD4^+ ^T lymphocyte depletion in *ex vivo*-cultures of human tonsil tissue [8,36] and provide the experimental framework to perform standardized multi-donor HLAC analyses and map Nef determinants that are critical for these activities.

**Figure 2:**
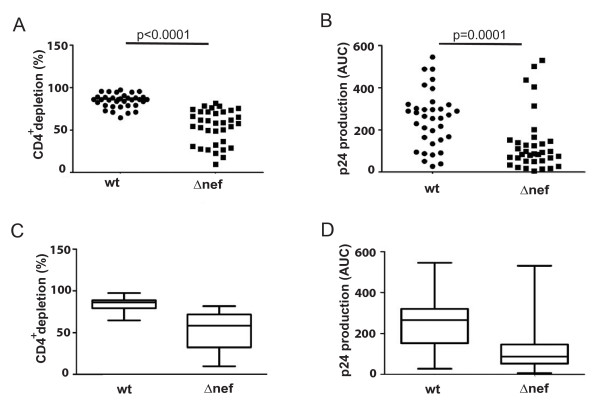
**CD4^+ ^T cell depletion and viral replication of wt and Δ*nef *HIV-1 in tonsils from 35 experiments**. (A, B) CD4^+ ^T cell depletion (A) and p24 production (B). Each data point represents the mean value of quadruplicate parallel infections. p-values indicate statistical significance by Mann-Whitney-U analysis. (C, D) Boxplot presentation of the data shown in A and B. Boxes indicate the upper and lower quartile (box borders), median (line within the box) and the maxima and minima of each data set (vertical lines).

**Figure 3:**
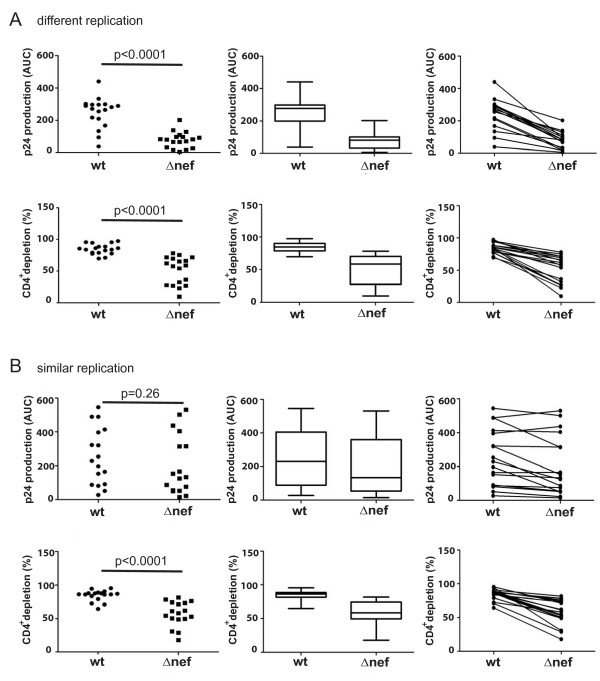
**CD4^+ ^T cell depletion and viral replication of wt and Δ*nef *HIV-1 in selected cohorts**. Experimental data analyzed in Fig. 2 were divided into two groups based on differences in p24 production established for wt and Δ*nef *viruses. Experiments, in which p24 production between wt and Δ*nef *HIV-1 differed significantly (p > 0.05) were grouped as "different replication" (A). Experiments without significant difference (p < 0.05) in p24 production were considered as "similar replication" (B). Shown are p24 production and CD4^+ ^T cell depletion of all individual experiments (left panels). p-values indicate statistical significance by Mann-Whitney-U analysis. The middle panels are boxplot presentations of the data on the left with the indicated upper and lower quartile, median, maxima and minima. In the right panels, data are presented to identify wt and *Δnef *pairs analyzed in parallel.

Using the experimental set-up described above, we first compared virus replication and CD4^+ ^T lymphocyte depletion of wt and *Δnef *HIV-1 using tonsils from 14 donors in 35 independent experiments, e.g. with independent virus stocks, with quadruplicate parallel infections for each time point analyzed. These studies demonstrated that CD4^+ ^T lymphocyte depletion was consistently less severe for the *nef*-deficient virus compared to the isogenic wt HIV-1 (wt: 84.4 ± 1.4% vs Δ*nef*: 53.8 ± 1.4%; p < 0.0001) (Fig. 2A, C). Similarly, p24 production and thus virus replication was also significantly reduced in the absence of Nef (wt: (mean) 251.1 ± 9.8 vs Δ*nef: *(mean) 134.1 ± 4.6; p = 0.0001) (Fig. 2B, D), suggesting, for this cross-donor analysis, a correlation between virus replication and loss of CD4^+ ^T lymphocytes. Upon closer examination of the results for HLAC from individual donors, we noted that while HIV-1*Δnef *depleted CD4^+ ^T lymphocytes less vigorously than an average infection with wt HIV-1 in essentially all experiments (Fig. 2A, C), p24 production by HIV-1*Δnef *reached levels in the range of the highest ones obtained with wt HIV-1, in some, but not all experiments (Fig. 2B). To explore this further, we stratified results obtained for individual donor HLAC according to p24 production into two groups: the first, for which AUC values for *Δnef *HIV-1 were statistically lower than those of wt (p < 0.05, 18 experiments; different replication; Fig. 3A), and the second, for which the AUC values did not reveal statistically significant differences between wt and *Δnef *HIV-1 (p ≥ 0.05, 17 experiments; similar replication; Fig. 3B). Expectedly, both p24 production and CD4^+ ^T lymphocyte depletion were markedly decreased in HIV-1 *Δnef*-infected relative to wt HIV-1-infected cultures in the "different replication" cohort (production: wt: 248.9 ± 13.4 vs *Δnef: *79.3 ± 5.7 (p < 0.0001); depletion: wt 84.5 ± 1.9% vs. *Δnef *50.7 ± 2.1% (p < 0.0001); mean values) (Fig. 3A). In the "similar replication" cohort p24 production was, expectedly, statistically indistinguishable between both viruses (wt: 253.0 ± 14.7 vs *Δnef: *202.1 ± 7.0; p = 0.26; mean values) (Fig. 3B), but wt HIV-1 infection still resulted in a more pronounced CD4^+ ^T lymphocyte depletion than HIV-1*Δnef *infection (wt: 84.4 ± 2.2% vs *Δnef: *57.2 ± 2.0%, mean values), with a high statistical significance (p < 0.0001). In line with these findings, no statistically significant correlation between HIV-1 replication and CD4^+ ^T lymphocyte depletion was observed (data not shown). These multi-donor studies demonstrate that Nef boosts HIV-1 replication and depletion of CD4^+ ^T lymphocytes in HLAC, and suggests that Nef's effect on the loss of CD4^+ ^T lymphocytes is not strictly coupled to the elevation of virus spread.

### Nef employs two distinct protein interaction surfaces to facilitate the depletion of CD4^+ ^T lymphocytes in HLAC

**Figure 4:**
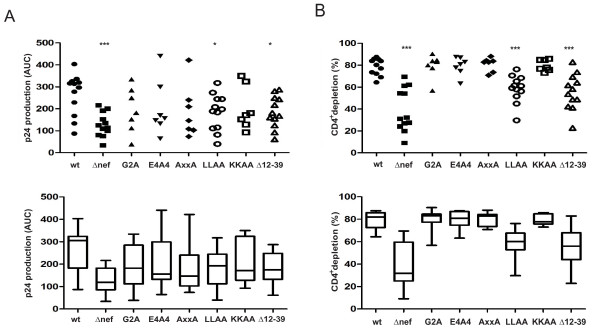
**Molecular determinants for Nef's activity in HIV-infected HLAC**. HLACs were infected with 3 ng p24 of wt HIV-1, *Δnef *HIV-1 or HIV-1 encoding for the indicated Nef mutants in quadruplicates. (A) p24 production over the culture period. (B) CD4^+ ^T cell depletion on day 12 p.i. Asterisks indicate statistical significance by Mann-Whitney-U analysis (*** p ≤ 0.0005, ** p < 0.005, * p < 0.05). The top panels present mean values of all individual experiments (n = 7–12), bottom panels are boxplots of the data shown on the top with the indicated upper and lower quartile, median, maxima and minima.

To determine the molecular determinants that govern Nef's activity in this *ex vivo*-model, tonsil aggregate cultures were challenged with a panel of isogenic HIV-1 NL4-3 viruses coding for characterized SF2 Nef variants [27]. Fig. 4 summarizes the results from up to 12 individual experiments. HIV-1 wt and *Δnef *served as reference controls and showed the expected and statistically highly significant difference in CD4^+ ^T cell depletion and p24 production. While all HIV-1 infections with the analyzed Nef mutants displayed apparently intermediate p24 production (Fig. 4A), this difference only reached low statistical significance for NefLLAA and NefΔ12–39 (Δ*nef *p = 0.0017, G2A p = 0.16, E4A4 p = 0.19, AxxA p = 0.12, LLAA p = 0.03, KKAA p = 0.33, Δ12–39 p = 0.026). When compared among them, virus production between these various Nef mutant viruses was statistically indistinguishable. Despite these comparable intermediate levels of virus production, specific Nef mutants significantly differed in their ability to deplete CD4^+ ^T lymphocytes (Fig. 4B). Four of the analyzed mutants were statistically indistinguishable in their depletion activity from wt HIV-1. This included the G2A and KKAA mutants that lack N-terminal myristoylation or membrane microdomain targeting signals, respectively, and thus display reduced membrane binding (G2A) or lack membrane microdomain incorporation (KKAA) (G2A: 79.5%, p = 0.93; KKAA: 79.9%, p = 1.0) [37]. The two other mutants, E4A4 and AxxA, are not disturbed in their subcellular localization [27], but lack protein interaction motifs for the phosphofurin acidic cluster sorting protein (PACS) sorting adaptor (E4A4) or SH3 domains (AxxA), and are deficient in modulating MHC class I cell surface levels and cell activation, respectively (E4A4: 79.1%, p = 0.899; AxxA: 80.9%, p = 0.93) (reviewed in [24,25]). The lack of requirement for these motifs suggested that these activities are dispensable for Nef-mediated CD4^+ ^T lymphocyte depletion in HLAC infections. In contrast, the two other Nef mutants LLAA and Δ12–39 were significantly impaired in CD4^+ ^T lymphocyte depletion, displaying only partial activity relative to wt (LLAA: 58.6%, p = 0.0002; Δ12–39: 55.6%, p = 0.0005). NefLLAA fails to interact with the endocytic machinery responsible to internalize CD4 and is thus defective in downregulating cell surface CD4 (reviewed in [38]). The Δ12–39 Nef variant, in contrast, is fully active in CD4 downregulation but lacks the interaction surface for the Nef-associated kinase complex (NAKC), a multiprotein complex that facilitates transcription of the HIV-1 genome [39,40]. These results reveal that Nef requires a complex set of molecular determinants to boost HIV-1 spread in HIV-1-infected HLAC and identify two independent protein interaction motifs in Nef that facilitate CD4^+ ^T lymphocyte depletion independently of their effect on HIV-1 replication.

### Nef enforces death of CD4^+ ^T cells in HIV-infected HLAC

**Figure 5:**
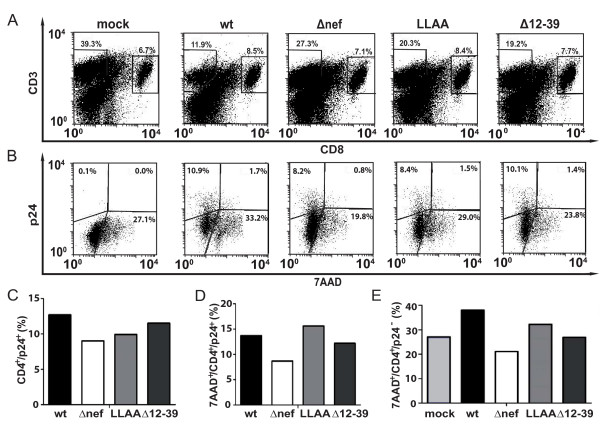
**Analysis of cell death in HIV-1-infected HLAC**. HLACs were infected with 6 ng p24 of wt HIV-1, *Δnef *HIV-1 and the two Nef variants LLAA and Δ12–39 in quadruplicates. On day 8 p.i., cells were stained for 7AAD, CD3, CD8 and intracellular p24. Results from one single infection are shown. (A) Flow cytometry analysis of the CD3/CD8 stain of live T lymphocytes. (B) Flow cytometry analysis of CD3^+^/CD8^- ^lymphocytes (based on the gating in A) for intracellular p24 and 7AAD for the detection of productively HIV-1 infected and dead CD4^+ ^T cells, respectively, (C) Percentage of total productively infected (p24^+^) CD4^+ ^lymphocytes in the HLAC infection shown in A. (D) Percentage of dead (7AAD^+^) and infected (p24^+^) CD4^+ ^T lymphocytes in the HLAC infection shown in A. (E) Percentage of dead (7AAD^+^) uninfected (p24^-^) CD4^+ ^T lymphocytes in the HLAC infection shown in A. Mock designates the percentage of dead CD4^+ ^T lymphocytes in the mock-infected culture.

**Figure 6:**
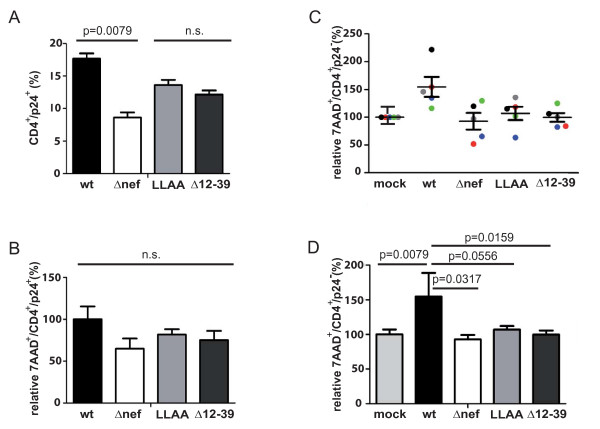
**Nef augments bystander T cell death in HIV-infected HLAC**. Summary of cell death analysis in HLAC infections as described in Fig. 5 from quadruplicate infections of HLAC from 5 donors. (A) Percentage of total productively infected CD4^+ ^T lymphocytes. (B) Percentage of 7AAD-positive infected CD4^+ ^T lymphocytes relative to wt that was arbitrarily set to 100%. (C, D) Percentage of dead uninfected CD4^+ ^T lymphocytes relative to mock controls that were arbitrarily set to 100%. C depicts individual data points with the colour code identifying separate experiments, D the mean and SEM of these experiments. p-values indicate statistical significance based on Mann-Whitney-U analysis.

To further analyze how Nef accelerates depletion of CD4^+ ^T lymphocytes in HLAC HIV-1 infection, we sought to determine whether Nef primarily affects the killing of productively infected or uninfected (bystander) CD4^+ ^T cells. Since reproducible analyses of specific markers for apoptosis such as active caspase 3 or cell surface annexinV in combination with the fixation method and intracellular stain for p24 proved difficult in our experimental system (data not shown), we analyzed cell death by staining with 7AAD, a nucleic acid dye that binds to genomic DNA of dead, necrotic and late apoptotic cells because of their increased membrane permeability. For analysis, we gated on CD4^+ ^T lymphocytes to determine their 7AAD incorporation and intracellular p24 expression (see Methods for details). Control experiments ensured that the 7AAD staining was stable during the fixation and permeabilization procedures (data not shown). The infection with wt HIV-1 was compared to Δ*nef *HIV-1 as well as HIV-1 coding for the Nef LLAA or Δ12–39 variants, the two mutants that displayed defects in CD4^+ ^T lymphocyte depletion in the above analyses. Fig. 5 shows primary data of such an analysis for one donor. As before, CD4^+ ^T lymphocytes were identified as CD3^+^/CD8^- ^population, carefully avoiding autofluorescent cells in the center of the dot plot (Fig. 5A). When these CD4^+ ^T lymphocytes were analyzed for the expression of p24 and staining for 7AAD (Fig. 5B), the mock-infected culture, expectedly, only showed background staining for p24. On day 8 p.i., 27.1% of all CD4^+ ^T lymphocytes stained positive for 7AAD and were thus considered dead. In wt HIV-1-infected cultures, a population of p24-positive viable CD4^+ ^T lymphocytes was readily detectable (10.9%, upper left quadrant). A small fraction of p24-positive cells also stained positive for 7AAD (1.7% of all CD4^+ ^T lymphocytes; 13.7% of all p24^+ ^CD4^+^T lymphocytes; upper right quadrant) (Fig. 5D). Notably, wt HIV-1 infection increased the number of 7AAD^+^/p24^- ^cells (33.2% of all CD4^+ ^T lymphocytes; 38.0% of all p24^- ^CD4^+ ^T lymphocytes; lower right quadrant), reflecting bystander killing in response to virus infection (Fig. 5E). This increase in death of p24^- ^bystander cells was less pronounced in cultures infected with HIV-1Δ*nef *(19.8% of all CD4^+ ^T lymphocytes; 21.1% of all p24^- ^CD4^+ ^T lymphocytes). Plotting of the total number of productively infected CD4^+ ^T lymphocytes revealed slightly fewer infected cells in cultures infected with the Δ*nef *relative to the wt virus (Fig. 5C). Interpretation of these results was complicated by the varying degree of HIV-unrelated cell death between HLAC replicates, but also HLAC cultures from different donors. To account for this, analysis of quadruplicate infections of HLAC from five different donors was performed to quantify and statistically evaluate this effect (Fig. 6). First, total amounts of p24^+ ^cells present in these cultures (irrespective of their sensitivity to staining with 7AAD) (Fig. 6A) were significantly increased in the presence of Nef (wt: 17.7 ± 0.8% vs Δ*nef: *8.6 ± 0.7%, p = 0.0079). Nef thus expands the pool of productively infected cells in HIV HLAC infections. Although the 7AAD staining appeared slightly increased in cells infected with wt HIV-1 relative to Δ*nef *or the two Nef mutants (Fig. 6B, data expressed as percentage relative to wt that was arbitrarily set to 100%), these differences were not statistically significant (wt: 100 ± 15.2%; Δ*nef*: 65.1 ± 11.7%; LLAA: 81.6 ± 6.3%; Δ12–39: 74.9 ± 11.0%). This indicated that Nef does not have major pro- or anti-apoptotic effects on infected CD4^+ ^T lymphocytes in HIV-infected HLAC. However, since HIV infection clearly reduces the overall life span of CD4^+ ^T lymphocytes [41], the fact that Nef expands the target cell population will indirectly contribute to T cell depletion in HLAC. In contrast, significantly more killing of bystander CD4^+ ^T lymphocytes (Fig. 6C and 6D) was observed in HIV-1 wt-infected cultures when compared to infections with the Δ*nef *or the LLAA and Δ12–39 Nef mutant viruses [wt: 154.6 ± 34.2% (p = 0.0079 compared to mock); Δ*nef*: 92.8 ± 6.5% p = 0.0317; LLAA: 107 ± 5.3% p = 0.0556; Δ12–39: 99.7 ± 5.9% p = 0.0159, data expressed as percentage relative to mock that was arbitrarily set to 100%]. These results identify the interaction motifs for endocytic machinery and the NAKC signalosome as key determinants for Nef-mediated depletion of CD4^+ ^T lymphocytes in HIV-1 HLAC infections and suggest that Nef selectively augments the efficacy of bystander killing.

## Discussion

This study aimed at the characterization of the role of Nef in HIV-1 replication and depletion of CD4^+ ^T lymphocytes in *ex vivo*-aggregate cultures of human tonsil tissue. Extensive comparison of wt and Δ*nef *HIV-1 in HLAC from a large number of donors demonstrated that Nef elevates the efficacy of both HIV spread and CD4^+ ^T lymphocyte depletion. Loss of CD4^+ ^T lymphocytes apparently occurred predominantly in uninfected bystander cells and was more pronounced in the presence of Nef even in scenarios where wt and Δ*nef *viruses displayed comparable replication kinetics and p24 production. A panel of isogenic Nef mutant viruses consistently revealed a partial segregation of Nef activities: while a large number of molecular determinants in Nef was required for optimal HIV-1 spread in HLAC, two distinct protein interaction surfaces were identified that specifically govern Nef-mediated enhancement of CD4^+ ^T lymphocyte depletion which preferentially occurs in bystander cells.

The results presented confirm and extend the previously observed enhancement of HIV-1 replication in HLH by Nef [8,36]. Typically, *nef*-positive viruses grow with faster kinetics and higher peak titers than their *nef*-negative isogenic counterparts. In line with earlier work, these results establish HLAC as a relatively robust assay system for effects of Nef on HIV-1 replication *ex vivo*. However, the magnitude of elevated HIV-1 spread observed in the presence of Nef is not markedly more pronounced than that obtained in PBMC cultures [27]. The overall effects of Nef in this *ex vivo*-cell system are significantly lower than the 2–3 log increase in virus titers reported *in vivo *[18]. This may, in part, reflect the lack of a CTL response to HIV-infected cells in *ex vivo *model systems, which is potently evaded by Nef *in vivo*, the relatively short time frame of the experiments as well as the use of a lab-adapted CXCR4 using HIV-1 strain. In contrast to PBMC cultures, however, HLAC allows a concurrent and prospective experimental analysis of CD4^+ ^T lymphocyte depletion induced by HIV-1 infection. Thus, HIV-infected HLAC recapitulates some of the key activities of Nef *in vivo*, but the magnitude of the effects is attenuated in this *ex vivo*-primary cell culture system.

Although we cannot fully exclude that Nef expression leads to elevated intracellular p24 levels, the fact that significantly less p24^+ ^cells are present in the absence of Nef suggests that the lack of Nef limits the infection of new target cells rather than affecting the amounts of particles produced per productively infected CD4^+ ^T lymphocyte. This would be in line with conclusions reached by Glushakova et al. that reported comparable levels of per cell HIV-1 gene expression in HLAC infections in the presence and absence of Nef [8]. Our analysis of specific molecular Nef determinants revealed a complex scenario in which each Nef mutant individually caused a reduction of p24 levels (HIV-1 production and spread) relative to the wt protein. While the precise effector functions of these individual protein surfaces in HLAC remain unclear, these results suggest that, similar to the scenario in PBMC cultures [27,42], several independent Nef activities are necessary, but not sufficient, for optimal HIV-1 replication.

Of note, comparison of p24 production between wt and *Δnef *HIV-1 in HLAC revealed comparable growth of both viruses in approximately one half of the experiments, while Nef promoted overall p24 production (Fig. 3) and replication kinetics (data not shown) in the other half of the experiments. As such divergent results were also obtained with identical virus stocks, they most likely reflect different properties of HLAC from different donors, suggesting that Nef's effect on HIV-1 spread *ex vivo *can be context-dependent. Similar replication kinetics of both viruses could in principle reflect a loss of Nef function or a circumvention for the need for Nef in these cells. Since HIV-1 replication in these cultures reached levels typically observed for wt HIV-1 in HLAC from other donors, we conclude that these target cells are preconditioned to support the efficient spread of *nef*-deleted HIV-1. An obvious difference between HLAC from individual donors may be the intrinsic activation state of the target CD4^+ ^T lymphocytes. However, extensive analysis of T cell activation markers including CD25, CD69 and HLA-DR prior and during HIV-1 infection failed to reveal any correlation between the expression of these markers and detection of a Nef-dependent increase of HIV-1 replication (data not shown). We also failed to observe major differences in surface levels of the entry coreceptor CXCR4 in mock infected HLAC from different donors, suggesting that the observed donor variability does not reflect altered permissivity for infection of these cultures due to changes in entry coreceptor cell surface exposure (data not shown). The identification of host cell conditions that desensitize cells for the Nef-mediated elevation of HIV-1 replication efficiency will be an important aim of future studies.

Importantly, we failed to detect significant effects of Nef on the frequency of dead HIV-infected, p24^+ ^CD4^+ ^T lymphocytes in HLAC, a primary cell model that displays robust HIV-mediated cytotoxicity. This was somewhat surprising given that Nef expression has been reported to cause both pro- as well as anti-apoptotic effects (e.g. [43-49]). However, most of these studies were performed in cell line overexpression systems instead of HIV-1 infected primary cells and did not involve analyses of HLAC cultures. Rather, our results agree with a recent report by Schindler *et al*. [50], in which no effect of Nef on apoptosis triggered by a variety of stimuli was observed in HIV-1-infected PBMC cultures. Jekle and colleagues established that depletion of CD4^+ ^T lymphocytes by HIV-1 infection in HLAC predominantly results from the killing of uninfected bystander cells [17]. Our attempts to verify these findings were complicated by the fact that the intracellular p24 staining procedure used most likely fails to sensitively detect infection of late apoptotic and thus proteolytic active cells, and 7AAD does not efficiently score for cells early in apoptosis with intact membranes. These technical limitations, however, equally apply to cells infected with all the viruses compared in this study; and the frequency of cell death in infected and bystander cells detected closely matched that of apoptotic cells detected by others [17]. Although populations of apoptotic and/or productively infected cells might have been missed, our results revealed a substantial amount of dead uninfected cells in HIV-1 infected HLAC, the proportion of which was increased in the presence of Nef. Collectively, our results therefore suggest that the Nef-mediated increase of CD4^+ ^T lymphocyte depletion predominately stems from the elevated death of p24-negative bystander cells.

The mapping of molecular determinants that govern Nef-mediated increase in CD4^+ ^T lymphocyte death identified the C-terminal di-leucine motif and the interaction site for the NAKC signalosome as two distinct protein interaction sites involved in this process. Individual mutation of both motifs significantly impaired Nef-mediated CD4^+ ^T lymphocyte depletion, indicating that both motifs are critical for this activity. Surprisingly, non-myristoylated Nef (G2A) retained wt activity in CD4^+ ^T lymphocyte depletion, although membrane association via its myristoylated N-terminus is thought to be vital for virtually all Nef activities. However, membrane association and biological activity such as CD4 downregulation of the Nef allele used here (HIV-1 SF2) is only partially impaired by the G2A mutation due to additional membrane targeting motifs [27,37], indicating that Nef induced CD4^+ ^T lymphocyte depletion can occur in the absence of myristoylation and thus only requires moderate membrane affinity. Analysis of the AxxA Nef mutant also revealed that interactions with SH3 domain containing ligands are dispensable for CD4^+ ^T cell depletion. In SIV-infected macaques, the relevance of the SH3-binding motif in Nef is controversial [51-53]. In *ex vivo *studies, however, this Nef protein interaction surface affects receptor transport (e.g. downregulation of cell surface MHC-I) as well as cell activation events (e.g. association with PAK2 kinase activity), two processes that do not correlate with Nef activity in HLAC [36,54]. This is well in line with the observed wt activities of the Nef E4A4 and KKAA mutants that are disrupted in the interaction site for PACS (involved in MHC-I downmodulation, [55]) and targeting to plasma membrane microdomains (where Nef associates with PAK2 [37,56]).

As mentioned above, we favor a scenario in which CD4^+ ^T lymphocyte depletion occurs predominately in uninfected bystander cells. The results presented allow us to speculate by which mechanism Nef might stimulate this process. Death of bystander CD4^+ ^T lymphoyctes in HIV-1 infected HLAC is induced by the shedding of Env glycoprotein from infected cells and the subsequent triggering of cell death programs upon interaction of soluble Env with CXCR4 on uninfected bystander cells [17]. Similarly, cell death could be triggered by cell-associated Env via direct contacts with neighboring cells. As a consequence of both mechanisms, the number of infected cells is one determining parameter for the efficacy of bystander apoptosis in the culture. One simple reason for the elevated levels of bystander cell death in infections with wt relative to Δ*nef *HIV-1 can thus be attributed to the larger pool of productively infected cells. However, the observed increase in bystander cell death in the absence of elevated replication levels in some HLACs together with the identification of molecular determinants that specifically govern Nef-mediated bystander killing suggest that Nef also exerts more direct effects. In one possible scenario, Nef promotes shedding of Env and subsequent bystander killing. In fact, by reducing tethering of Env to the primary entry receptor CD4 at the surface of HIV-producing cells, downmodulation of cell surface CD4 by Nef increases the levels of Env in released virions [57-59]. Via the same mechanism, Nef might facilitate shedding of Env from the cell surface, an activity that would be hampered by disruption of CD4 downmodulation via the LLAA mutation. Nef also promotes plasma membrane exposure of Env via a CD4-independent mechanism [60], an effect that may well be supported by enhanced transcriptional activity of the viral genome due to the assembly of NAKC [39,40]. Thus, both identified determinants for Nef-mediated bystander killing could synergize to maximum cell surface presentation and shedding of Env which is prone to trigger bystander killing. Although only tested herein in the context of a CXCR4 using HIV-1 strain, Nef generally exerts its activities with comparable efficiency in the context of viruses that use CCR5 as an entry coreceptor [61]. However, the cytotoxicity of CCR5 tropic Env proteins is relatively reduced, explaining why CD4^+ ^T lymphocyte depletion is generally mild and can only be slightly enhanced by Nef in HLAC infections with R5-tropic strains [9,62]. Finally, Nef was recently implicated in the cell surface regulation of the program death receptor PD-1 in infected cells [63,64]. Analyzing Nef variants from SIV infected sooty mangabeys, Schindler and colleagues could correlate the potency by which Nef prevents PD-1 surface exposure *ex vivo *with low levels of CD4 T lymphocyte loss *in vivo*, while HIV-1 Nef variants failed to downregulate cell surface PD-1 levels [64]. As PD-1 can induce death in bystander cells expressing PD-1 ligands, it will be of interest to analyze the role of PD-1 in Nef-mediated bystander cell death in HLH infections.

## Conclusion

Together, this analysis of Nef function in HLAC revealed that Nef promotes the depletion of CD4^+ ^T lymphocytes by two mechanisms. First, Nef expands the pool of productively infected cells, thereby elevating net levels of cell killing. Second, Nef increases the intrinsic potential of such productively infected cells to trigger killing, presumably most prominently in uninfected bystander cells. These results underscore the usefulness of HLAC as an experimental system for the *ex vivo*-analysis of pathogenic processes in HIV-1 infection and suggest that triggered bystander killing may contribute to Nef's pathological properties in infected patients.

## Methods

### Virus constructs and stocks

All proviral plasmids used here were previously described and the expressed Nef variants characterized [27,37]. Briefly, *nef *genes and thus also the overlapping U3 enhancer/promoter region from the HIV-1_SF2 _(wild-type or carrying the indicated mutations) were inserted into the HIV-1_NL4-3 _proviral DNA and compared to a *nef*-deleted variant entirely lacking Nef expression. All Nef variants analyzed herein express to comparable levels in HIV-1 infected T lymphocytes [27,37]. Virus stocks were generated by transfection of proviral HIV plasmids into 293 T cells as described [65]. Two days after transfection, culture supernatants were harvested. The HIV-1 p24 antigen concentration of concentrated stocks was determined by a p24 antigen enzyme-linked immunosorbent assay (ELISA) [66].

### Human Lymphoid Aggregate Culture (HLAC) from tonsil

Tonsil tissue was removed during routine tonsillectomy from HIV-, HBV-, HCV-negative patients with informed consent. To prepare HLAC, tonsil tissue was mechanically dispersed by cutting tissue in 2- to 3-mm blocks and passing them through 40-μm cell strainers (BD Falcon, Belgium). Cells were washed in PBS, and 2 × 10^6 ^cells were plated in 96-well V-bottom plates (Corning Incorporated, New York, NY) in a final volume of 200 μl. Culture medium (RPMI 1640 containing 15% fetal bovine serum, 1% L-glutamine, 1% fungizone, 1% gentamycin (all from GIBCO), 0.25% ampicillin (Roth, Karlsruhe, Germany), 1% non-essential amino acids, and 1% sodium pyruvat (both from Invitrogen)). Detailed cultivation methods have been reported [12,17]. One day after tonsil preparation, the HLAC was inoculated with HIV-1 (3–6 ng p24 per 2 × 10^6 ^cells per well). Following overnight infection cells were washed and the culture medium was subsequently changed every three days without dispersing the pellet. At the same time intervals supernatant samples were harvested and stored at -20°C for subsequent analysis by p24 ELISA.

### Flow cytometry for CD4^+ ^T lymphocyte depletion and cell death analysis

All flow cytometry was performed with a FACS Calibur with BD CellQuest Pro 4.0.2 Software (BD Pharmingen). For the analysis of CD4^+ ^T lymphocyte depletion, cells were stained with the following antibodies without prior permeabilization: anti-human CD3 FITC (clone HIT3a, BD Pharmingen), anti-human CD8 APC (clone RPA-T8, BD Pharmingen) and subsequently fixed (1.5 hrs, 2% paraformaldehyde). Using standard gating procedures [17], CD4^+ ^T lymphocytes were then defined as the CD3^+^/CD8^- ^lymphocyte population. To determine the death of HIV-infected and non-infected CD4^+ ^T lymphocytes, cells were first stained for anti-human CD3 APC (clone HIT3a, BD Pharmingen) and anti-human CD8 PE (clone RPA-T8, BD Pharmingen) and 7AAD (2.5 μg/ml) (BD Pharmingen) as described above. Following subsequent fixation cells were permeabilized in PBS with 0.1% Triton X-100 and simultaneously stained for intracellular p24 using anti-p24 antibody KC57 FITC (Beckman Coulter, Fullerton, CA) 20 min at RT.

### Statistical analysis

Analysis of HLAC infections was carried out in independent quadruplicate infections for each time point investigated. Mean values and standard deviation were calculated from these quadruplicates. For comparison of individual HLAC infections, mean values of each data set were averaged with the indicated standard error of the mean. For evaluation of statistical significance, data sets were first analyzed for normal distribution by the Kolmogorov-Smirnow test. As this test for Normality failed for some populations, the non-parametric Mann-Whitney-U analysis was employed throughout (***, P ≤ 0.0005; **, P < 0.005; *, P < 0.05). GraphPad Prism software (GraphPad Software Inc.) was used for all statistical analyses.

For the analysis of CD4^+ ^T cell depletion, the ratio of CD4^+ ^to CD8^+ ^T lymphocytes was determined and expressed relative to the uninfected control whose ratio was arbitrarily set to 100%. Depletion efficiencies were calculated relative to this value with the CD4^+ ^to CD8^+ ^ratio of uninfected cultures considered as 0% depletion. For quantification of virus replication, p24 concentrations of individual infections were plotted over the course of the experiment. To account best for rapid changes in p24 concentration, the integral area under the curve (AUC) was determined and used as measure of overall p24 production. To determine the percentage of killed infected and non-infected bystander CD4^+ ^T cells, four populations of CD4^+ ^T lymphocytes were defined and quantified: uninfected, living cells (p24^-^/7AAD^-^), uninfected, dead cells (p24^-^/7AAD^+^), infected, living cells (p24^+^/7AAD^-^) and infected, dead cells (p24^+^/7AAD^+^).

## Competing interests

The authors declare that they have no competing interests.

## Authors' contributions

SH and NT performed the experimental work. OTK and OTF conceived the experimental strategies and SH, OTK and OTF designed individual experiments. IB and SS provided tonsillectomy material. SH, OTK and OTF analyzed the data and OTF wrote the manuscript.

## Supplementary Material

Additional File 1 **Supplementary figure one.** Direct staining of CD4 was avoided due to the reduction of CD4 surface exposure in HIV-1 infected cells, but a control staining for mock infected cells reveals that virtually all CD3^+^/CD8^- ^cells in this gate were positive for CD4. Click here for file
