# Supplementary figures and images for: Determinants in HIV-1 Nef for enhancement of virus replication and depletion of CD4+ T lymphocytes in human lymphoid tissue ex vivo
Source: Retrovirology. 2009 Jan 15;6:6. doi: 10.1186/1742-4690-6-6 (PMC2630989; doi:10.1186/1742-4690-6-6)

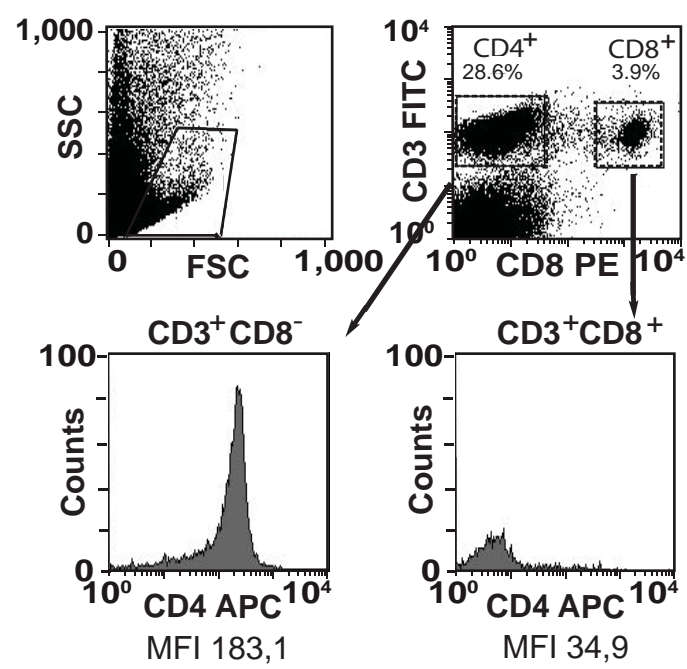

Supplemental Figure 1 Homann et al.

Supplement: Additional File 1 — Supplementary figure one. Direct staining of CD4 was avoided due to the reduction of CD4 surface exposure in HIV-1 infected cells, but a control staining for mock infected cells reveals that virtually all CD3+/CD8- cells in this gate were positive for CD4. [file 1742-4690-6-6-S1.pdf]
